# Comparison of lipid accumulation product and visceral adiposity index with traditional obesity indices in early-onset type 2 diabetes prediction: a cross-sectional study

**DOI:** 10.1186/s13098-023-01056-3

**Published:** 2023-05-27

**Authors:** Chen-Ying Lin, Jing-Bo Li, Fan Wu, Jun-Jia Wang, Hao-Hua An, Hui-Na Qiu, Long-Fei Xia, Yao-Shuang Li, Ya-Jie Zhai, Chun-Jun Li, Jing-Na Lin

**Affiliations:** 1 Tianjin Union Medical Center, Tianjin Medical University, Tianjin, China; 2 Department of Endocrinology, Health Management Center, Tianjin Union Medical Center, Nankai University Affiliated Hospital, No. 190, Jieyuan Rd, Hongqiao District, Tianjin, 300121 China; 3 School of Medicine, Nankai University, Tianjin, China; 4 Graduate School, Tianjin University of Traditional Chinese Medicine, Tianjin, China; 5 Department of Clinical Laboratory, Tianjin Union Medical Center, Nankai University Affiliated Hospital, Tianjin, China

**Keywords:** Early-onset type 2 diabetes, Lipid accumulation product, Visceral adiposity index, Body mass index, Waist circumference, Obesity, Young adult, Chinese

## Abstract

**Background:**

The purpose of the study was to compare the efficacy of two novel obesity indices, lipid accumulation product (LAP) and visceral adiposity index (VAI), with traditional obesity indices in predicting early-onset type 2 diabetes (T2DM).

**Methods:**

In this cross-sectional study, a total of 744 participants, including 605 patients newly diagnosed with T2DM and 139 non-diabetic control subjects, were enrolled from a tertiary care hospital in Tianjin, China. Participants with T2DM were divided into two groups based on their age at diagnosis, namely early-onset T2DM (age less than 40 years, n = 154) and late-onset T2DM (age 40 years or older, n = 451). The predictive power of each obesity index was evaluated using receiver operating characteristic (ROC) curve analysis. Furthermore, binary logistic regression analysis was conducted to examine the independent relationship between LAP and VAI with early-onset T2DM risk. The relationship between novel obesity indices and the age of T2DM onset was also evaluated through correlation and multiple linear regression analysis.

**Results:**

In males, LAP had the highest predictive power for early-onset T2DM with an area under the ROC curve (AUC) of 0.742 (95% CI 0.684–0.799, P < 0.001). In females, VAI had the highest AUC for early-onset T2DM with a value of 0.748 (95% CI 0.657–0.839, P < 0.001), which was superior to traditional indices. Patients in the 4th quartile of LAP and VAI had 2.257 (95% CI 1.116–4.563, P = 0.023) and 4.705 (95% CI 2.132–10.384, P < 0.001) times higher risk of T2DM before age 40, compared to those in the 1st quartile, respectively. A tenfold increase in LAP was associated with a decrease in T2DM onset age of 12.862 years in males (β = −12.862, P < 0.001) and 6.507 years in females (β = −6.507, P = 0.013). A similar decrease in T2DM onset age was observed for each tenfold increase in VAI in both male (β = −15.222, P < 0.001) and female (β = −12.511, P < 0.001) participants.

**Conclusions:**

In young Chinese individuals, LAP and VAI are recommended over traditional obesity indices for improved prediction of early-onset T2DM risk.

## Introduction

Type 2 diabetes mellitus (T2DM) has traditionally been regarded as a disease affecting middle-aged and older adults, however, the trend of increasing prevalence among young adults, adolescents, and children in China is a cause for alarm [1, 2]. A recent national cross-sectional study in China revealed that 36% of individuals between the ages of 18–39 years had diabetes or pre-diabetes [3]. Early-onset T2DM patients, defined as those diagnosed before the age of 40, have been shown to have poorer metabolic control [4, 5], a higher risk of chronic complications, and elevated all-cause mortality [6]. The early onset of T2DM places a significant economic and psychological burden on young adults [7] and is particularly concerning for young females during their childbearing years, as it increases the likelihood of exposure to hyperglycemic environments for their offspring [8]. Thus, there is an urgent need to implement screening measures to identify and control T2DM risk factors in order to delay its onset.

Obesity has been identified as a significant and modifiable risk factor for T2DM in young adults. Studies have demonstrated that body mass index (BMI) is inversely related to the age of T2DM diagnosis [9]. The National Surveys on Students' Constitution and Health (NSSCH) data shows that the incidence of overweight and obesity in children and adolescents has increased 16-fold from 1985 to 2014, pointing to an increasing prevalence of early-onset T2DM in China in the future [10]. Abnormal fat distribution, rather than total fat mass, is a crucial factor in the development of metabolic disorders [11, 12]. Visceral adipose tissue (VAT) is particularly harmful and has been shown to be an independent predictor of T2DM risk [13]. Chinese adults tend to have higher levels of visceral fat compared to white populations with the same BMI [14]. A study in China revealed that the prevalence of central obesity among Chinese adults reached 40.9% in 2013–14 [10]. A study from the SEARCH also indicated that a high waist circumference (WC) is particularly prominent in youth with T2DM, with a prevalence of 95% [15]. These findings suggest that visceral fat deposition may play a crucial role in the risk of early onset T2DM in young Chinese. Hypertriglyceridemia, resulting from high VAT lipolysis, has also been noted in younger T2DM patients, who have shown to have higher triglyceride (TG) levels and lower high-density lipoprotein cholesterol (HDL-C) levels compared to older T2DM patients [5, 12, 16–18]. A longitudinal observational study from the Swedish National Diabetes Register found that these differences persist several years after diagnosis [16].

In 2005, Kahn [19] introduced a new index called the "Lipid Accumulation Product" (LAP), which is a combination of waist circumference and fasting plasma triglyceride levels for determining lipid buildup and predicting excessive visceral obesity in adults. In 2010, Amato and colleagues [20] developed a novel, sex-specific obesity index, the "Visceral Adiposity Index" (VAI), based on waist circumference, body mass index, triglyceride levels, and high-density lipoprotein cholesterol levels^.^ Previous studies have shown that both LAP and VAI are strong predictors of T2DM [21, 22]. Given that individuals with early-onset T2DM have a higher likelihood of having abdominal obesity, elevated triglyceride levels, and reduced high-density lipoprotein cholesterol levels, it is hypothesized that LAP and VAI may increase the risk of early-onset T2DM. The purpose of this study is to examine the association between the novel obesity indices LAP and VAI and early-onset T2DM and to compare the predictive abilities of LAP and VAI with traditional obesity indices for early-onset T2DM.

## Materials and methods

### Study population

The study included 726 newly diagnosed diabetic inpatients with a duration of less than 1 year who were admitted to the endocrine department at Tianjin Union Medical Center between January 2019 and June 2022. Inclusion in the study was based on the diagnostic criteria for diabetes mellitus outlined by the World Health Organization (WHO) in 1999 [23]. Excluded from the study were patients under the age of 18, those with type 1 diabetes mellitus, gestational diabetes, or other special forms of diabetes, and those with an unclear type of diabetes. The classification of diabetes mellitus was also based on the WHO consultation [23]. Additionally, participants with ketosis, serious infections, malignant tumors, serious heart, liver, kidney, or thyroid dysfunction, and those taking lipid-lowering drugs or thiazide diuretics daily for the past month were also excluded. The exclusion criteria were established to eliminate potential confounding factors that may impact the lipid profiles of the participants. Furthermore, individuals with incomplete key variables such as anthropometric measurements and blood lipid profile results were excluded.

A total of 49 participants were excluded due to diabetic ketosis and/or T1DM and/or severe infections. One individual with steroid-induced diabetes, one with pancreatogenic diabetes, five with malignant tumors, and seven with severe cardiac, hepatic, and renal damage were also excluded from the study. Additionally, 53 participants were not included in the analysis due to insufficient information on key variables, and an additional five outliers were removed for the purposes of accurate analysis. The final sample comprised 605 individuals, who were divided into two groups: early-onset T2DM and late-onset T2DM, based on their age. The control group comprised 139 healthy individuals who were matched for age and gender to the early-onset T2DM group and underwent a routine physical examination at the Health Management Center of Tianjin Union Medical Center. All subjects in the control group were confirmed as non-diabetic through fasting blood glucose and glycosylated hemoglobin tests.

### Data collection

Clinical data was collected through interviews conducted by health professionals, such as doctors or postgraduate medical students. The interviews covered information related to general characteristics (e.g. gender, age, date of diabetes diagnosis, and duration of diabetes), lifestyle habits (e.g. smoking and alcohol consumption), and medical history (e.g. family history of diabetes, previous illnesses, and medication use). Family history of diabetes was defined as the presence of diabetes in at least one first-degree relative (parents, siblings, or children). Smoking status was classified as either current or non-current, with current smokers defined as those who smoke daily and non-current smokers including those who have never smoked, occasionally smoked, or quit. Drinking status was similarly categorized as current or non-current, with current drinkers defined as those who drink daily and non-current drinkers including those who have never drunk, occasionally drank, or quit.

In our study, height and weight were measured using an automatic height and weight measuring instrument (DST-600, DONGHUAYUAN, China) by health professional surveyors. The WC was measured utilizing a soft ruler at the midpoint between the lower ribs and the iliac crest. Blood pressure was measured with an arm-type electronic sphygmomanometer (AC-05C, Ling Qian, China) after participants had rested for at least 5 min. The visceral fat area (VFA) and body fat ratio (BFR) were assessed through bioelectrical impedance analysis (BIA) using a body composition analyzer (InBody770, Biospace, Korea). The presence of fatty liver was primarily determined through transabdominal ultrasound. All diabetic patients underwent an oral glucose tolerance test (OGTT). The participants fasted overnight for at least 8 h, and venous blood samples were collected the following morning for laboratory examination.TG, total cholesterol (TC), HDL-C, low-density lipoprotein cholesterol (LDL-C), uric acid (UA), and fasting plasma glucose (FPG) were measured using an automatic biochemical analyzer (TBA-120FR, Toshiba, Japan). The levels of insulin and C-peptide were determined through radioimmunoassay, and the GHbA1c was detected through high-efficiency liquid chromatography utilizing an automatic glycosylated hemoglobin analyzer (HA-8180, ARKRAY, Japan).

Ultimately, the clinical data were extracted from the electronic medical records by designated recorders who meticulously recorded the relevant information on a pre-determined form. To maintain impartiality, the interviewers, surveyors, and recorders were not informed of the study's underlying hypothesis. Two impartial assessors were also blinded to the exposure status of the study participants and their sole involvement in the study was the evaluation of the results.

### Definition and calculation of variables

We used the homeostasis model assessment-2(HOMA2) model to calculate the homeostasis model assessment-2 of insulin resistance (HOMA2-IR) and the homeostasis model assessment-2 of β-cell function (HOMA2-β). The HOMA2 model was obtained from www.ocdem.ox.ac.uk [24]. The HOMA2 model was used after converting the units of C-peptide to nmol/L and inputting the fasting glucose and fasting C-peptide values into the model, in order to avoid the effect of exogenous insulin. In addition, the following indices were calculated and defined:

BMI = body weight (kg)/the square of the height (m^2^) [25].

LAP = [WC (cm)-65] × TG (mmol/L) for males; LAP = [WC (cm)-58] × TG (mmol/L) for females [19].

VAI = $\left(\frac{\text{WC}}{39.68+\left(1.88\times BMI\right)}\right)\times \left(\frac{TG}{1.03}\right)\times \left(\frac{1.31}{HDL}\right)$ for males; VAI = $\left(\frac{\text{WC}}{36.58+\left(1.89\times BMI\right)}\right)\times \left(\frac{TG}{0.81}\right)\times \left(\frac{1.52}{HDL}\right)$ for females; WC (cm), BMI (kg/m^2^), TG (mmol/L) and HDL (mmol/L) [20].

### Statistical analysis

Normally distributed and skewed data approximated a normal distribution after natural log transformation (including SBP, DBP, HOMA2-IR, HOMA2-β, TG, HDL-C, VFA, VAI and LAP) were compared by Student's t-test. We compared categorical variables between the two groups by the chi-square test.

In order to determine the predictive ability of the LAP and VAI in comparison to other traditional obesity-related indices (BMI, WC, BFR, and VFA) for early-onset T2DM, we employed a receiver operating characteristic (ROC) curve analysis. This analysis involved plotting ROC curves, which utilized early-onset T2DM as the state variable, late-onset T2DM as the reference variable, and LAP, VAI, BMI, WC, BFR, and VFA as the test variables. The evaluation of the results consisted of calculating the area under the curve (AUC), cut-off value, sensitivity, specificity, and the Youden index.

To evaluate the correlation between various levels of LAP and VAI with the risk of early-onset T2DM, we divided the sample into four subgroups using the quartile divisions of LAP (L1-L4) and VAI (V1-V4). The quartile categorization of LAP in males was L1 (≤ 30.29), L2 (30.30–51.35), L3 (51.36–93.49), and L4 (> 93.51), while VAI was classified as V1 (≤ 1.36), V2 (1.37–2.16), V3 (2.17–3.62), and V4 (> 3.63). In females, the quartile categorization of LAP was L1 (≤ 30.90), L2 (30.91–52.90), L3 (52.91–81.34), and L4 (> 81.35), and that of VAI was V1 (≤ 1.71), V2 (1.72–2.76), V3 (2.77–4.28), and V4 (> 4.29). Subsequently, a binary logistic regression analysis was executed, with L1 or V1 as the reference, to calculate the adjusted OR and 95% confidence intervals (95% CI) of the other subgroups after combining the quartiles of both genders. To assess the independent influence of LAP and VAI on early-onset T2DM, variables with a significance level (α) of less than 0.1 from univariate analysis were included in the model, after adjusting for multicollinearity. The original model of LAP consisted of variables such as current drinking status, smoking status, systolic blood pressure (SBP), diastolic blood pressure (DBP), UA, fatty liver, family history of diabetes, LDL-C, HDL-C, and LAP quartile categorization. Similarly, the original model of VAI incorporated variables such as drinking status, smoking status, SBP, DBP, UA, fatty liver, family history of diabetes, LDL-C, and VAI quartiles. The final models were obtained using forward stepwise regression.

Finally, in an effort to provide a clearer comprehension of the impact of LAP and VAI on the onset age of T2DM by gender, we conducted a correlation analysis and multiple linear regression after log-transforming LAP and VAI using base 10 logarithm. Pearson's correlation analysis was employed in the correlation analysis, while multiple linear regression was utilized to establish predictive models for the onset age of T2DM. A stepwise model selection approach was employed, evaluating the goodness of model fit using the Akaike Information Criterion (AIC). We carried out standard linear regression diagnostics, including verification of linearity, normality, and homoscedasticity of residuals, as well as checking for the absence of leverage points and outliers.

We regarded *P* < 0.05 (two-tailed) to be statistically significant. The statistical analysis was carried out utilizing RStudio (version 2021.09.0 for Mac OS, RStudio Team, RStudio: Integrated Development for R; RStudio, Inc., Boston, MA).

## Results

### Clinical and biochemical characteristics of research subjects

Out Of the 744 subjects, 154 (20.7%) were diagnosed with early-onset T2DM, 451 (60.6%) with late-onset T2DM, and the remaining 139 (18.7%) served as the control group. The clinical and biochemical profiles of the three groups are depicted in Table 1. The mean age of diagnosis for the early-onset group was 34.4 years [standard deviation (SD) = 4.7], while it was 59.1 years (SD = 8.2) for the late-onset group. A higher proportion of male patients (76.6%) was observed in the early-onset group, with significantly higher levels of uric acid and HOMA2-IR compared to the late-onset group (all *P* < 0.05). The prevalence of current smoking was also significantly higher among individuals with early-onset T2DM, compared to the late-onset and control groups (*P* < 0.05). No differences in current drinking status were noted among the three groups. The early-onset group also had a higher prevalence of a family history of diabetes (60.4%), fatty liver (74.7%), and higher DBP and SBP compared to the late-onset and control groups (all *P* < 0.05). Notably, the early-onset group had significantly elevated levels of TG and lower levels of HDL-C compared to the late-onset and control groups (all *P* < 0.001).

**Table 1:** Comparison of clinical and biochemical characteristics between the three groups

Variables	Early-onset (n = 154)	Late-onset (n = 451)	*P* value	Control group (n = 139)	*P*^†^ value
Age (years)	34.4 (4.7)	59.1 (8.2)	< 0.001^***^	33.1 (4.6)	0.035^*^
Male gender (%)	118 (76.6)	244 (54.1)	< 0.001^***^	107 (77.0)	0.943
Family history of diabetes (%)	93 (60.4)	201 (44.6)	0.001^**^	29 (20.9)	< 0.001^***^
Current smoker (%)	43 (27.9)	86 (19.1)	0.021^*^	25 (18)	0.044^*^
Current drinker (%)	24 (15.6)	97 (21.5)	0.113	15 (10.8)	0.228
SBP (mmHg)	132.0 (120.0–145.0)	130.0 (120.0–140.0)	0.037^*^	124.0 (113.0–131.0)	< 0.001^***^
DBP (mmHg)	84.0 (80.0–94.5)	80.0 (76.0–85.0)	< 0.001^***^	75.0 (68.0–82.0)	< 0.001^***^
Fatty liver (%)	115 (74.7)	219 (48.6)	< 0.001^***^	64 (46.0)	< 0.001^***^
HbA1C (mmol/mol, %)	10.1 (2.0)	9.8 (2.3)	0.079	5.4 (0.3)	< 0.001^***^
HOMA2-IR	2.3 (1.6–3.0)	2.1 (1.5–2.8)	0.034^*^		
HOMA2-β	53 (33.9–80.6)	47.7 (32.1–71.5)	0.038^*^		
TG (mmol/L)	2.4 (1.6–3.9)	1.6 (1.2–2.3)	< 0.001^***^	1.2 (0.8–1.7)	< 0.001^***^
TC (mmol/L)	5.0 (1.2)	5.0 (1.1)	0.618	4.9 (0.9)	0.778
HDL-C (mmol/L)	1.0 (0.9–1.1)	1.1 (1.0–1.3)	< 0.001^***^	1.3 (1.1–1.5)	< 0.001^***^
LDL-C (mmol/L)	3.3 (1.0)	3.2 (0.8)	0.130	3.1 (0.7)	0.041^*^
UA (μmol/L)	342.4 (88.1)	284.2 (79.6)	< 0.001^***^	365.3 (86.9)	0.053

*T2DM* type 2 diabetes mellitus, *SD* standard deviation, *TC* total cholesterol, *LDL-C* low-density lipoprotein cholesterol, *UA* uric acid, *IQR* interquartile range, *SBP/DBP* systolic/diastolic blood pressure, *HOMA2-IR/β* homeostasis model assessment-2 of insulin resistance/β cell function, *TG* triglyceride, *HDL-C* high-density lipoprotein cholesterol

^*^P < 0.05; **P < 0.01. ***P < 0.001

^†^P-value between the control group and early-onset group; Data are expressed as mean (SD) for normally distributed variables; median (IQR, Q25-Q75) for skewed variables; or frequency (percentage, %) for categorical variables. P values were derived from Student's t-test or chi-square test

### Comparison of obesity-related indices

**Fig. 1:**
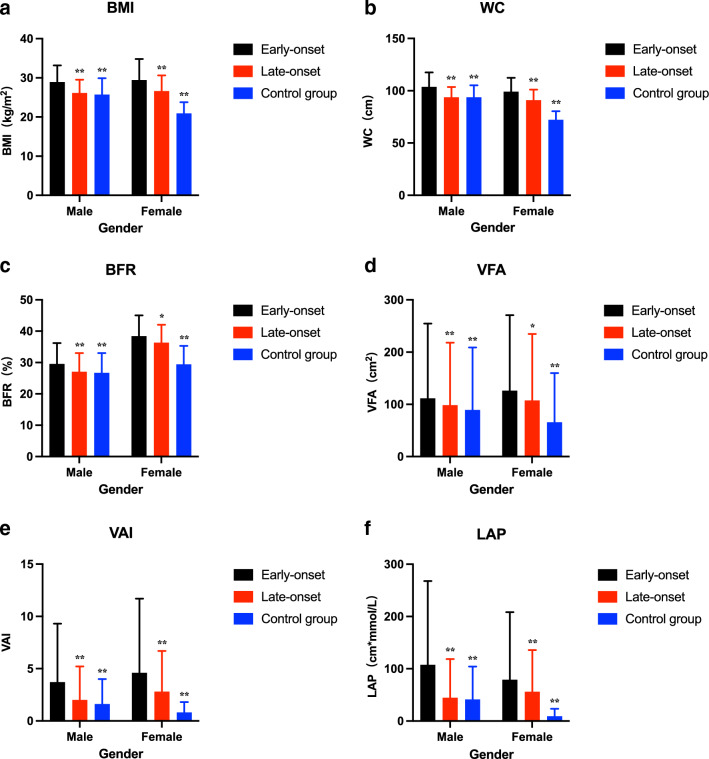
Comparison of obesity-related indices between the three groups. **a** BMI, body mass index; **b** WC, waist circumference; **c** BFR, body fat ratio; **d** VFA, visceral fat area; **e** VAI, visceral adiposity index; **f** LAP, lipid accumulation product. Column graph with error bar: top of the column represents the mean (**a**, **b**, **c**) or median (**d**, **e**, **f**); the error bar represents SD (**a**, **b**, **c**) or interquartile range (**d**, **e**, **f**). ^∗^*P* < 0.05, ^∗∗^*P* < 0.01; all *P* values for Student's t-test

As depicted in Fig. 1, among both male and female subjects, all of the obesity-related indices in the study were significantly higher in the early-onset group than in the late-onset and control groups (all *P* < 0.05). The mean BMI for evaluating overall obesity was found to be higher in the early-onset group compared to the late-onset and control groups in both males (28.9 vs. 26.1 vs. 25.7 kg/m^2^, all P < 0.001) and females (29.4 vs. 26.6 vs. 20.9 kg/m^2^, all P < 0.001). Furthermore, the study showed that patients in the early-onset group had significantly higher levels of WC (103.7 vs. 93.7 vs. 93.6 cm, all *P* < 0.001 in males; 99.1 vs. 90.9 vs. 72.2 cm, all *P* < 0.001 in females), BFR (29.5% vs. 27% vs. 26.7% cm^2^, all *P* < 0.001 in males; 38.4% vs. 36.3% vs. 29.4%, all *P* < 0.05 in females), and VFA (111.3 vs. 98.4 vs. 89.2 cm^2^, all *P* < 0.001 in males; 126.0 vs. 107.3 vs. 65.5 cm^2^, all *P* < 0.05 in females) compared to the late-onset and control groups. Additionally, the early-onset group displayed significantly higher levels of novel obesity-related indices, including the VAI [3.7(2.1–5.6) vs. 2(1.3–3.2) vs. 1.6(0.9–2.4), all *P* < 0.001 in males; 4.6(3.1–7.1) vs. 2.8(1.9–3.9) vs. 0.8(0.7–1), all *P* < 0.001 in females] and LAP [107.3(52.5–160.6) vs. 44.3(29.6–74.2) vs. 41(20.1–63.1) cm_*_mmol/L, *P* < 0.001 in males; 78.9(54.2–129.5) vs. 55.8(35.4–80) vs. 9.2(5.9–14.2) cm_*_mmol/L, *P* < 0.001 in females], compared to the late-onset and control groups.

### The performance of obesity-related indices in predicting early-onset T2DM

**Fig. 2:**
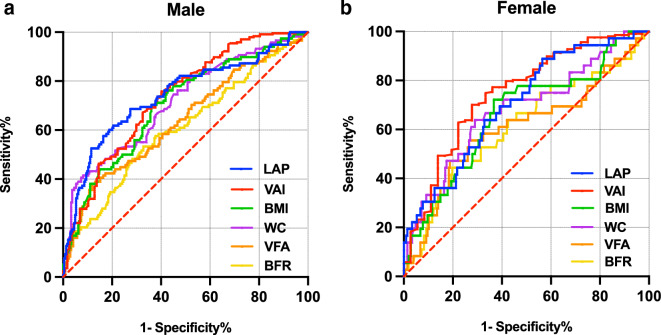
ROC curves of obesity-related indices for predicting early-onset T2DM by gender. **a** ROC curves of obesity-related indices in males; **b** ROC curves of obesity-related indices in females. *LAP* lipid accumulation product, *VAI* visceral adiposity index, *BMI* body mass index, *WC* waist circumference, *VFA* visceral fat area, *BFR* body fat ratio

As depicted in Fig. 2 and Table 2, the results of the ROC curve analysis evaluated the predictive ability of obesity-related indices for early-onset T2DM. In male subjects, six indices, LAP, VAI, WC, BMI, VFA, and BFR were all found to be predictive of early-onset T2DM (all *P* < 0.001). The AUC values ranged from 0.607 to 0.742, with LAP exhibiting the highest AUC of 0.742. In female subjects, five indices, VAI, LAP, WC, BMI, and BFR were found to be predictive of early-onset T2DM, with the highest AUC of 0.748 observed for VAI (all *P* < 0.05). Notably, both of LAP and VAI exhibited higher AUC values than traditional obesity indices, such as WC, BMI, VFA, and BFR, when predicting the onset of early-onset T2DM in both male and female subjects. LAP had AUC values of 0.742 in males and 0.705 in females, while VAI had AUC values of 0.733 in males and 0.748 in females. In contrast, the AUC values for traditional obesity indices ranged from 0.607 to 0.714 in males and 0.613 to 0.680 in females. Our DeLong test revealed that only VFA and BFR demonstrated a statistically significant difference in AUC when compared to LAP and VAI in males (LAP vs. VFA P = 0.0001; LAP vs. BFR, P < 0.0001; VAI vs. VFA P = 0.0033; VAI vs. BFR P = 0.0003). In contrast, no statistically significant differences in AUC were observed across various obesity-related indices in females. These findings suggest that LAP and VAI are potentially more efficient predictors of early-onset T2DM, especially in males.

**Table 2:** ROC analysis of obesity-related indices for predicting early-onset T2DM

Indices	AUC (95% CI)	Cut-off	Sens. (%)	Spec. (%)	Youden Index	*P* value
Male						
LAP	0.742 (0.684–0.799)	> 81.46	61.86	79.51	0.4137	< 0.001^***^
VAI	0.733 (0.677–0.789)	> 3.21	59.83	75.82	0.3565	< 0.001^***^
WC	0.714 (0.656–0.772)	> 106.1	41.53	91.80	0.3333	< 0.001^***^
BMI	0.702 (0.644–0.759)	> 26.89	70.34	63.52	0.3386	< 0.001^***^
^#^VFA	0.639 (0.576–0.701)	> 133.36	38.14	88.11	0.2625	< 0.001^***^
^#^BFR	0.607 (0.544–0.671)	> 29.54	53.39	67.21	0.2060	< 0.001^***^
Female						
VAI	0.748 (0.657–0.839)	> 3.59	72.22	70.05	0.4227	< 0.001^***^
LAP	0.705 (0.615–0.795)	> 47.85	88.89	43.48	0.3237	< 0.001^***^
WC	0.680 (0.579–0.782)	> 95.2	63.89	71.01	0.3490	< 0.001^***^
BMI	0.663 (0.562–0.763)	> 27.37	72.22	63.29	0.3551	0.0016^**^
BFR	0.613 (0.505–0.721)	> 40.46	44.44	80.19	0.2464	0.0409^*^
VFA	0.607 (0.495–0.720)	> 124.2	55.56	73.43	0.2899	0.0615

*ROC* receiver operating characteristic, *AUC* areas under the ROC curve, *CI* confidence interval, *Sens* sensitivity, *Spec* specificity, *LAP* lipid accumulation product, *VAI* visceral adiposity index, *WC* waist circumference, *BMI* body mass index, *VFA* visceral fat area, *BFR* body fat ratio

^#^Statistically significant differences were observed between the AUC of the novel indices LAP and VAI and other traditional obesity-related indicators (P values for the DeLong test were < 0.05)

^*^P < 0.05; ^**^P < 0.01; ^***^P < 0.001

### Association of novel obesity indices with early-onset T2DM

**Fig. 3:**
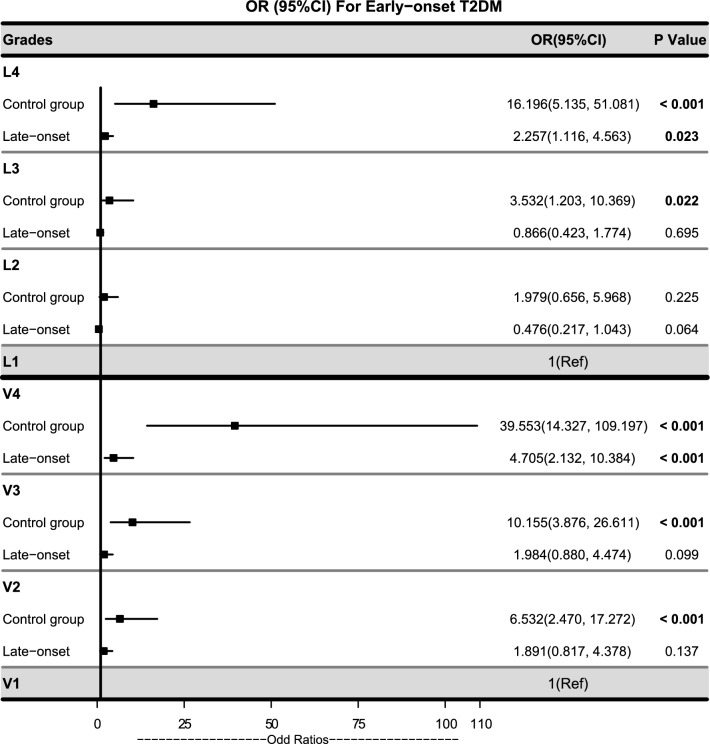
ORs and 95% CIs for early-onset T2DM of LAP quartiles and VAI quartiles. *OR* odds ratio, *CI* confidence interval, *L1-L4* quartile 1–4 levels of LAP, *V1-V4* quartile 1–4 levels of VAI. The models of LAP quartiles were adjusted for the current drinker and smoker status, SBP, DBP, UA, fatty liver, family history of diabetes, LDL-C, and HDL-C. The models of VAI quartiles were adjusted for the current drinker and smoker status, SBP, DBP, UA, fatty liver, family history of diabetes, and LDL-C

The adjusted odds ratios (ORs) for early-onset T2DM, along with 95% confidence intervals (CIs), in the L2-L4 and V2-V4 subgroups were determined by binary logistic regression, using the lowest quartile of the LAP and VAI as the reference, as illustrated in Fig. 3. As compared to the control subjects, the risk of early-onset T2DM showed a significant increase with the elevation of LAP levels, starting from 1.979-fold (95% CI 0.656–5.968) in the L2 subgroup, to 16.196-fold (95% CI 5.135–51.081) in the L4 subgroup. Patients in the fourth quartile of LAP had a 2.257-fold (95% CI 1.116–4.563, *P* = 0.023) higher risk of developing T2DM prior to the age of 40, compared to those in the first quartile of LAP. Similar observations were made for VAI, where the risk of early-onset T2DM escalated from 6.532-fold in the V2 subgroup, to 10.155-fold in the V3 subgroup, and reached 39.553-fold in the V4 subgroup (all *P* < 0.001). The V4 subgroup patients had a 4.705-fold (95% CI 2.132–10.384, *P* < 0.001) higher risk of developing T2DM before the age of 40, as compared to the V1 subgroup patients.

### Relationship between age at onset of T2DM and novel obesity indices by gender

**Fig. 4:**
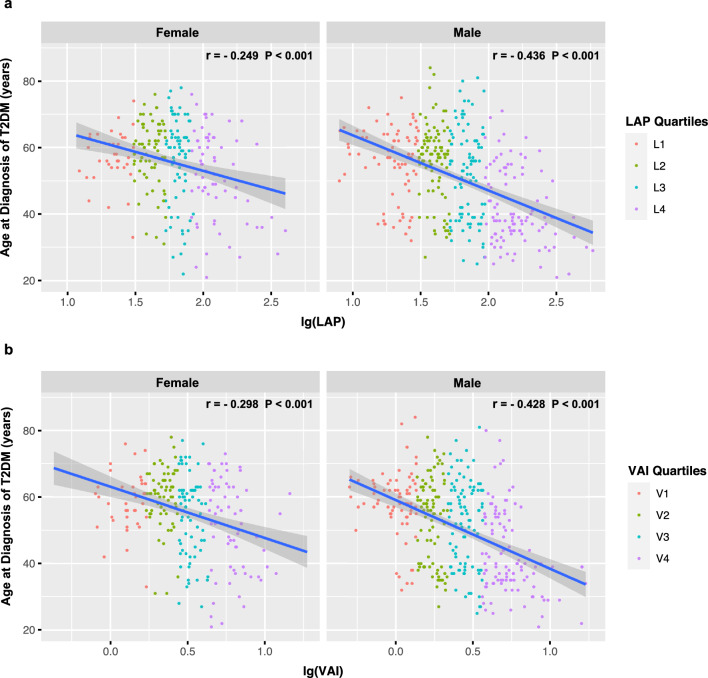
Relationship between novel obesity indices and age at T2DM onset. **a** Scatterplot between log10(LAP) and age of onset by gender; **b** scatterplot between log10(VAI) and age of onset by gender

The findings of the correlation analysis were presented in Fig. 4. A significant inverse relationship was detected between log10 (LAP) and age at the onset of T2DM in both males (r =−0.436, *P* < 0.001) and females (r =−0.249, *P* < 0.001). Furthermore, a strong negative correlation was also noted between log10 (VAI) and age at the onset of T2DM in both males (r =−0.428, *P* < 0.001) and females (r =−0.298, *P* < 0.001). Results from the multiple linear regression models, based on gender, were presented in Table 3. The results of the stepwise multiple regression analysis indicate that both LAP and VAI were independent predictors of age at T2DM onset. An increment of tenfold in LAP was associated with a decrease of 12.862 years in the age of onset of T2DM in males (β = −12.862, *P* < 0.001) and 6.507 years in females (β =−6.507, *P* = 0.013). Additionally, a tenfold increment in VAI was found to significantly decrease the age at onset of T2DM by 15.222 years in males (β = −15.222, *P* < 0.001) and by 12.511 years in females (β = −12.511, *P* < 0.001).

**Table 3:** Multiple linear regression models for age at diagnosis of T2DM by gender

Variables	Male		Female	
	Standardized coefficients(β)	*P* value	Standardized coefficients(β)	*P* value
log10(LAP)	−12.862 (−16.503, −9.220)	< 0.001^***^	−6.507 (−11.608, −1.405)	0.013^*^
Family history	−3.252 (−5.674, 0.829)	0.008^**^	−3.687 (−6.432, −0.941)	0.008^**^
Current drinker	1.961 (−0.734, 4.656)	0.153	−12.692 (−19.839, −5.545)	< 0.001^***^
Fatty liver	−2.009 (−4.594, 0.577)	0.127	−3.404 (−6.375, −0.433)	0.024^*^
DBP	−0.168 (−0.283, −0.052)	0.004^**^	−0.143 (6.986, 17.684)	0.063
HDL-C	4.720 (0.425, 9.015)	0.031^*^	12.335 (6.986, 17.684)	< 0.001^***^
LDL-C	−1.581 (−3.045, −0.117)	0.034^*^	–	–
log10(VAI)	−15.222 (−19.976, −10.470)	< 0.001^***^	−12.511 (−18.075, −6.947)	< 0.001^***^
Family history	−2.736 (−5.160, −0.312)	0.027^*^	−3.790 (−6.594, −0.986)	0.008^**^
Current drinker	2.117 (−0.521, 4.754)	0.115	−11.154 (−18.401, −3.906)	0.003^**^
Fatty liver	−2.640 (−5.177, −0.103)	0.041^*^	−3.822 (−6.809, −0.835)	0.012^*^
DBP	−0.189 (−0.303, −0.073)	0.001^**^	−0.164 (−0.317, −0.010)	0.036^*^
LDL-C	−1.479 (−2.904, −0.052)	0.042^*^	–	–
UA	−0.011 (−0.026, 0.004)	0.161	–	–

*LAP* lipid accumulation product, *VAI* visceral adiposity index, *DBP* diastolic blood pressure, *HDL-C* high-density lipoprotein cholesterol, *LDL-C* low-density lipoprotein cholesterol, *UA* uric acid

^*^P < 0.05; ^**^P < 0.01; ^***^ *P* < 0.001; *P* values were derived from F-test

## Discussion

### Early-onset T2DM and obesity

Cumulative exposure to obesity in young individuals has been shown to persistently reduce the age of diagnosis for T2DM [26]. This is further supported by a cross-sectional study of 999 Han Chinese T2DM patients in Taiwan, which revealed that adiponectin gene polymorphism was significantly associated with the age of T2DM diagnosis, suggesting that obesity may play a key role in elevating the risk of early-onset T2DM [27]. In a recent national population-based study conducted in Israel, Twig and colleagues observed an inverse relationship between BMI and the mean age of T2DM diagnosis [9]. Our study also supports these findings, LAP and VAI were found to have a significant negative correlation with the age at diagnosis of T2DM.

### LAP and early-onset T2DM risk

The LAP, a new obesity-related index, reflects the comprehensive changes in visceral fat accumulation in terms of anatomy and biochemistry [19]. In a 5-year prospective cohort study, Yan et al. found that the relative risk of developing diabetes was 1.95 (95% CI 1.41–2.70) and 2.20 (95% CI 1.12–4.30) in medium and high LAP trajectories, respectively, compared to low-trajectory LAP [28]. Similarly, a large cross-sectional study of 215,651 Chinese adults by Tian et al. reported that the prevalence of diabetes was significantly higher in the fourth quartile of LAP (21.76%) compared to the first quartile of LAP (5.72%) [21]. These findings are supported by a systematic review and meta-analysis of 18 studies by Khanmohammadi et al., which concluded that there is a strong association between LAP and T2DM [29]. Consistent with previous studies, we found that patients in the fourth quartile of LAP had a 16.196-fold higher risk of early-onset T2DM than those in the first quartile, with control subjects as reference. Furthermore, LAP was found to be a strong predictor of T2DM diagnosis before the age of 40, with patients in the fourth quartile having a 2.257-fold higher risk than those in the first quartile. These results are in line with previous studies, such as the Tehran Lipid and Glucose Study [30], which found that LAP was a better predictor of diabetes in younger individuals, and the study by Wakabayashi et al. [31], which found that the risk of diabetes associated with high LAP levels decreased with increasing age in both sexes.

### VAI and early-onset T2DM risk

The visceral adiposity index (VAI) is a novel index that integrates WC, BMI, TG, and HDL-C to reflect the abnormal distribution of visceral adipose [20, 32, 33] In a large-scale cross-sectional study of 11,113 rural Chinese individuals, WC and elevated levels of the ratio of TG to HDL-C were found to be strongly associated with T2DM [34]. Our study also revealed that patients with early-onset T2DM had higher WC and significantly higher levels of TG and lower levels of HDL-C compared to control and late-onset groups. Furthermore, multiple prospective cohort studies from Chinese populations have found a positive association between high levels of VAI and an increased risk of T2DM [22, 35–37]. A study by Yu and Yi et al. [37] showed that the risk of T2DM was significantly lower in the high-to-low VAI group compared to the maintained high VAI group (HR = 0.77, all *P* < 0.05). Our results showed that the risk of early-onset T2DM increased progressively with elevated VAI levels, with ORs ranging from 6.532, 10.155 to 39.553 (all* P* < 0.001) compared to normal controls. Furthermore, the fourth quartile of VAI was found to significantly increase the risk of pre-40 diagnosis in T2DM patients (OR 4.705, 95% CI 2.132–10.384). These findings are consistent with a recent systematic review of seven studies mostly from Chinese populations by Nusrianto et al. [38], which indicated that VAI is an independent predictor for T2DM, with ORs or HRs ranging from 1.2 to 3.6.

### Comparison of LAP and VAI with traditional obesity indices

The limitations of BMI and WC in differentiating between lean and fat mass and abdominal subcutaneous and visceral adipose tissue, respectively [39], led to a search for more effective obesity-related indices to predict the risk of early-onset T2DM. Our study found that LAP and VAI outperformed other obesity indices in the prediction of early-onset T2DM for both genders. In males, LAP demonstrated the highest AUC of 0.742, followed by VAI, WC, BMI, VFA, and BFR. For females, VAI had the highest AUC of 0.748, followed by LAP, WC, BMI, BFR, and VFA. An analysis by Kahn [40] of NHANES III data showed that in the fourth quartile, the OR of LAP in predicting diabetes was more than twice that of BMI, and LAP was more effective in predicting diabetes than BMI. Additionally, the Tehran lipid and glucose study [30] showed that LAP was a stronger predictor of diabetes than BMI. A systematic review by Khanmohammadi et al. [29] found that most studies showed that LAP was more effective in predicting T2DM than traditional indices, including BMI and WC. Several studies [35, 38, 41, 42] have demonstrated that VAI outperformed conventional indices (BMI, WC) in the discrimination of diabetes. In addition, Amato et al. [43] conducted a study that revealed that VAI is a robust index of visceral adipose tissue dysfunction in patients with T2DM, outperforming conventional obesity indices such as BMI, WC, and waist-to-hip ratio. However, Zhang et al. [36] reported that the AUC of VAI was not higher than that of other obesity indices (BMI, WC, waist-to-hip ratio, and waist-to-height ratio). Contrarily, a cohort study by Wang et al. [22] found that WC was the most powerful predictor of diabetes, with an AUC of 0.701, while VAI had the lowest AUC of 0.649.

Limitations of this study include a small sample size and limited generalizability to different populations. The study was conducted in a tertiary hospital with a homogeneous population. Further research is needed to validate these findings in larger and more diverse populations. Additionally, it would be valuable to explore the utility of LAP and VAI over a longer follow-up period to assess their long-term predictive accuracy.

In summary, early-onset T2DM was a more devastating phenotype with poorer metabolic control, which makes prevention of the premature onset of T2DM important. Both elevated LAP and VAI were strongly associated with increased risk for early-onset T2DM among young Chinese adults. Furthermore, LAP and VAI may be more effective predictors of T2DM onset before the age of 40 compared to traditional obesity indices such as WC, BMI, BFR, and VFA. Further studies are necessary to investigate the long-term effects of LAP and VAI on T2DM onset.

## Data Availability

The datasets used and/or analysed during the current study are available from the corresponding author on reasonable request.

## Abbreviations

T2DM: Type 2 diabetes mellitus
BMI: Body mass index
NSSCH: National Surveys on Students' Constitution and Health
VAT: Visceral adipose tissue
WC: Waist circumference
TG: Triglyceride
HDL-C: High-density lipoprotein cholesterol
LAP: Lipid accumulation product
VAI: Visceral adiposity index
WHO: World Health Organization
VFA: Visceral fat area
BFR: Body fat ratio
BIA: Bioelectrical impedance analysis
TC: Total cholesterol
LDL-C: Low-density lipoprotein cholesterol
UA: Uric acid
FPG: Fasting plasma glucose
HOMA2-IR: Homeostasis model assessment-2 of insulin resistance
HOMA2-β: Homeostasis model assessment-2 of β-cell function
ROC: Receiver operating characteristic curve analysis
AUC: Area under the curve
SBP: Systolic blood pressure
DBP: Diastolic blood pressure
AIC: Akaike information criterion
OR: Odds ratio
CI: Confidence interval
